# Ocular manifestations of COVID-19: A systematic review of current evidence

**DOI:** 10.1016/j.pmedr.2024.102608

**Published:** 2024-01-14

**Authors:** SeyedAhmad SeyedAlinaghi, Esmaeil Mehraeen, Arian Afzalian, Mohsen Dashti, Afsaneh Ghasemzadeh, Ava Pashaei, Amir Masoud Afsahi, Seyed Saeed Tamehri Zadeh, Iman Amiri Fard, AmirMohammad Vafaee, Ayoob Molla, Ramin Shahidi, Ali Dadjou, Mohammad Amin Habibi, Pegah Mirzapour, Omid Dadras

**Affiliations:** a Iranian Research Center for HIV/AIDS, Iranian Institute for Reduction of High Risk Behaviors, Tehran University of Medical Sciences, Tehran, Iran; b Department of Health Information Technology, Khalkhal University of Medical Sciences, Khalkhal, Iran; c School of Medicine, Tehran University of Medical Sciences, Tehran, Iran; d Department of Radiology, Tabriz University of Medical Sciences, Tabriz, Iran; e School of Nursing, University of British Columbia, Vancouver, British Columbia, Canada; f Department of Radiology, School of Medicine, University of California, San Diego (UCSD), CA, USA; g MSc Student in Geriatric Nursing, Department of Community Health Nursing and Geriatric Nursing, School of Nursing and Midwifery, Iran University of Medical Sciences, Tehran, Iran; h School of Medicine, Bushehr University of Medical Sciences, Bushehr, Iran; i Clinical Research Development Center, Qom University of Medical Sciences, Qom, Iran; j Department of Global Public Health and Primary Care, University of Bergen, Norway

**Keywords:** COVID-19, Ocular manifestations, Ophthalmic complication, Eye involvement

## Abstract

**Introduction:**

COVID-19 caused by SARS-CoV-2, commonly presents with symptoms such as fever and shortness of breath but can also affect other organs. There is growing evidence pointing to potential eye complications. In this article, we aim to systematically review the ocular manifestations of COVID-19.

**Methods:**

We conducted a systematic review to explore the ocular manifestations of COVID-19. We searched online databases including PubMed, Embase, Scopus, and Web of Science up to September 4, 2023. After a two-stage screening process and applying inclusion/exclusion criteria, eligible articles were advanced to the data extraction phase. The PRISMA checklist and Newcastle-Ottawa Scale (NOS) were used for quality and bias risk assessments.

**Results:**

We selected and extracted data from 42 articles. Most of the studies were cross-sectional (n = 33), with the highest number conducted in Turkey (n = 10). The most frequent ocular manifestation was conjunctivitis, reported in 24 articles, followed by photophobia, burning, chemosis, itching, and ocular pain. Most studies reported complete recovery from these manifestations; however, one study mentioned visual loss in two patients.

**Conclusion:**

In general, ocular manifestations of COVID-19 appear to resolve either spontaneously or with supportive treatments. For more severe cases, both medical treatment and surgery have been employed, with the outcomes suggesting that complete recoveries are attainable.

## 1 Introduction

Coronavirus disease 19 (COVID-19) is caused by Severe Acute Respiratory Syndrome Coronavirus 2)SARS-CoV-2(virus, first identified in Wuhan, China, in December 2019 (Nora et al., 2020). The disease quickly spread globally and was declared a pandemic on March 11, 2020 (Khalili et al., 2020). The rapid spread of COVID-19 had devastating consequences worldwide. Mortality rates varied, with approximately 80 % of hospitalized patients and 60 % of patients in the intensive care unit (ICU) surviving (Bedford et al., 2020).

The most common symptoms in COVID-19 are fever and shortness of breath (Wiersinga et al., 2020). While the virus primarily targets the lungs and immune system, it can also damage other organs, with the severity varying among patients (Chen and Jungang, 2020). COVID-19 complicates the clinical diagnosis process due to the absence of unique pathognomonic symptoms (Jin et al., 2020). Additionally, some patients may present with gastrointestinal (Han et al., 2020), renal and even ocular symptoms (Renu et al., 2020). The eye is an organ that plays a very important role in the transmission process of COVID-19, and protecting the eyes and doing this correctly during the epidemic is a must. On the other hand, it should be taken into account that sometimes eye manifestations can be the first or even the only manifestation of COVID-19, sometimes eye manifestations are a set of symptoms that can show themselves at any stage of the disease or even after recovery (Domínguez-Varela et al., 2021, Qu et al., 2021).

Most studies have emphasized respiratory symptoms, yet there is evidence pointing to potential eye complications (Seah and Agrawal, 2020). Research indicates that keratoconjunctivitis (Hutama et al., 2022), conjunctivitis (Chen et al., 2020, Maychuk et al., 2020, Wu et al., 2020), conjunctival irritation, diplopia and cotton wool patches are among the most prevalent ophthalmic symptoms in COVID-19 patients (Khan et al., 2021). Notably, viral conjunctivitis has been observed to affect men more frequently than women (Al-Namaeh, 2021). In some instances, these ophthalmic symptoms have been among the initial clinical manifestations of COVID-19 (Baig, 2020). Most reported cases were linked to acute manifestations of COVID-19, although some chronic instances have also been documented (Mahayana et al., 2020). Understanding the frequency and nature of ocular symptoms related to COVID-19 can assist physicians in making more accurate and timely diagnoses. This study underscores the significance of recognizing the prevalence and ocular manifestations of COVID-19 to facilitate better and earlier detection of the disease. Hence, we aim to systematically examine the ocular manifestations of COVID-19 in this article.

## 2 Methods

In this systematic review, we provide a comprehensive overview of the ocular manifestations associated with COVID-19 infection, drawing from the current literature. The authenticity and reliability of our findings are enhanced through adherence to the Preferred Reporting Items for Systematic Reviews and Meta-Analyses (PRISMA) checklist. Bias risk is also minimized by exploiting the Newcastle-Ottawa Scale (NOS) bias assessment tool.

### 2.1 Data sources

We searched for specified keywords and their combinations in the online databases of PubMed, Embase, Scopus, and Web of Science. All literature published in English up until September 4, 2023, were harvested for further investigations and data extraction processes. Below are the examples of keywords and their combinations used in our database searches:

A. “COVID-19” OR “SARS-CoV-2” OR “coronavirus disease 2019” OR “severe acute respiratory syndrome coronavirus 2” [Title/Abstract]
B. “Ocular” OR “Ophthalmic” [Title/ Abstract]
C. [A] AND [B]

### 2.2 Study selection

Screening and selection of the retrieved publications occurred in two steps. In the initial step, the titles and abstracts were assessed. In this step, four researchers (R.S., A.M., A.D., M.A.H.) identified relevant articles based on their titles and abstracts to be considered for the subsequent, more detailed stage. In this second stage, five other researchers (A.P., I.A.F., S.S.T.Z., P.M., A.M.V.) scrutinized the full texts of the screened articles and proceeded with extraction of necessary data.

Included publications met the following criteria: they were being original article, case series, or case reports, published in English, and underwent peer review process before publication.

We excluded the papers that met any of the following criteria: non-human such as animal or in vitro studies, experiments with insufficient published data, publications without accessible full texts, conference abstracts, duplicated papers, and editorial letters.

### 2.3 Data extraction

Five researchers (A.P., I.A.F., S.S.T.Z., P.M., A.M.V.) carried out extraction of essential data. This step was undertaken after the completion of the second selection phase, during which full texts were thoroughly reviewed. The extracted data is presented in Table 2. Additional checks for possible duplicate papers and extracted data were performed by other research members.

**Table 1:** Bias Risk Assessment of Included Studies Based on Newcastle-Ottawa Scale (NOS).

**Author (year)**	**Selection (out of 4)**	**Comparability (out of 2)**	**Exposure/Outcome (out of 3)**	**Total (out of 9)**
Giampietro et al. (2023)	***	*	***	**7**
Firat and Kobat (2021)	****	**	***	**9**
Ganesh and Mohanan-Earatt (2022)	***	*	***	**7**
Shaikh et al. (2022)	**	**	*	**6**
Sharma et al. (2022)	***	**	***	**8**
Shen et al. (2021)	**	**	***	**7**
Silveira et al. (2022)	**	**	***	**7**
Sindhuja et al. (2020)	**	*	*	**4**
Stephan et al. (2021)	****	**	***	**9**
Meduri et al. (2020)	***	**	**	**7**
Oncul et al. (2021)	***	**	***	**8**
Oren et al. (2021)	**	**	***	**7**
Oren and Kocabas (2022)	***	**	***	**8**
Perez-Chimal et al. (2021)	****	*	***	**8**
Pirraglia et al. (2020)	***	**	***	**8**
Ranzenigo et al. (2021)	****	**	***	**9**
Riotto et al. (2022)	***	**	**	**7**
Rodriguez-Ares et al. (2021)	***	**	**	**7**
Rokohl et al. (2020)	****	**	**	**8**
Kumar et al. (2021)	***	*	***	**7**
Ma et al. (2020)	***	**	***	**8**
Jiang et al. (2021)	***	**	***	**8**
Hepokur et al. (2023)	***	**	***	**8**
Zhou et al. (2020)	***	**	***	**8**
Wang et al. (2021)	***	**	***	**8**
Dag Seker and Erbahceci Timur (2021)	***	**	***	**8**
Layikh et al. (2021)	***	**	***	**8**
Sehgal et al. (2021)	****	*	**	**7**
Reinhold et al. (2021)	***	**	**	**7**
Jidigam et al. (2022)	***	**	**	**7**
Wan et al. (2022)	***	**	***	**8**
Bayram et al. (2022)	***	**	***	**8**
Abdelkader et al. (2021)	**	**	***	**7**
Abrishami et al. (2020)	***	**	***	**8**
Akturk Acar et al. (2022)	***	*	***	**7**
Ahuja et al. (2020)	****	**	***	**9**
Sezgin Akcay et al. (2021)	**	**	***	**7**
Boz et al. (2021)	***	**	***	**8**
Bypareddy et al. (2021)	***	**	***	**8**
Chen et al. (2020)	***	**	***	**8**
Scalinci and Trovato (2020)	***	**	***	**8**
Gangaputra and Patel (2020)	***	**	**	**7**

**Table 2:** Description of the findings reported in eligible studies.

**Author (year)**	**Country**	**Population**	**Comorbidity** **(%)**	**Ocular Manifestations (%)**	**Time of onset**	**Treatment of ocular Manifestations**	**Outcome**	**Other results**
Giampietro et al. (2023)	Brazil	150 patients	Hypertension (Samaranayake et al., 2020) HIV (Domínguez-Varela et al., 2021) Obesity (Khan et al., 2021) Diabetes mellitus (Ranzenigo et al., 2021)	Candle flame hemorrhage and cotton wool exudates (Qu et al., 2021)	N/A	N/A	N/A	N/A
Firat and Kobat (2021)	Turkey	32 patients 32 healthy	N/A	No significant differences in terms of central choroidal thickness, central foveal thickness, nasal 500, nasal 1500, temporal 500, or temporal 500 µm distances were found between the groups.	N/A	N/A	N/A	Choroidal and retinal thicknesses were not affected in patients with recent mild COVID 19 without comorbidities.
Ganesh and Mohanan-Earatt (2022)	India	13 patients	N/A	Anterior uveitis (Han et al., 2020) intermediate uveitis (Bedford et al., 2020) posterior uveitis (Nora et al., 2020) panuveitis (Khalili et al., 2020)	Within the 6 weeks after COVID-19 diagnosis	Topical Steroids (anterior uveitis) Topical, Oral, and IV Steroids, Mycophenolate mofetil, and MTX (intermediate, posterior, and panuveitis)	All patients responded well to treatment. Two patients had surgical treatment	Bilateral eye involvement (Renu et al., 2020) unilateral eye involvement (Chen and Jungang, 2020)
Shaikh et al. (2022)	Qatar	39 patients	Hypertension, diabetes mellitus, coronary artery disease, rheumatic arthritis, or cancer	Conjunctival hyperemia (33.3) eye pain (23.1) epiphora (20.5) burning sensation (10.3) photophobia (5.1) Conjunctivitis (5.1)	N/A	N/A	N/A	no correlation between patients’ gender, comorbidities, and occurrence of ocular manifestations in COVID-19 patients
Sharma et al. (2022)	India	658 (total) 162 (ocular)	Diabetes mellitus followed by hypertension (18.42)	Lid swelling (8.64) Watering and irritation (25.30) Follicular conjunctivitis (7.40) blephritis, lid hyperemia, dry eye, foreign body sensation, photophobia, chemosis and blurring of the vision and *peri*-orbital pain	mostly < 1 week after COVID-19 infection	N/A	56.7 % relieved from ocular discomfort	The most significant ocular morbidity was black discoloration of lids and peri ocular skin, lid swelling, and redness and purulent discharge of conjunctivitis needed emergency ophthalmic reference
Shen et al. (2021)	China	Total 3198 Ocular 28	hypertension (28.6), Glaucoma (3.6), Nephritis (3.6), autoimmune anemia (3.6), diabetes (3.6), hepatitis B (3.6), cerebral infarction (3.6)	Conjunctivitis (100 %)	N/A	Levofloxacin eye drop (15 = 53.6 %) Ganciclovir eye drop (2 = 7.1 %) sodium hyaluronate eye drop (1 = 3.6 %) artificial tears (1 = 3.6 %) observe (9 = 32.1 %)	N/A	conjunctivitis was a rare and self-limited complication in adults with COVID-19 while the existence of coronavirus receptors on human ocular surface and mouse lacrimal glands indicated the risk of SARS-CoV-2 infection
Silveira et al. (2022)	Brazil	Total 104 Ocular 36	N/A	Burning (19.23), pain (11.54), foreign body sensation (7.7), hyperemia (7.7), and tearing (3.84)	concomitantly with general symptoms (77.7) one day before flu-like symptoms (11.11 %) after 3 days(11.11 %)	eye drops for selfmedication (ketorolac trometamol and carmellose sodium)(5.71 %)	N/A	
Sindhuja et al. (2020)	India	Total 127 Ocular 12	Diabetes mellitus (Seah and Agrawal, 2020), hypertension (Renu et al., 2020), thyroid disorders (Khalili et al., 2020), pulmonary tuberculosis (Nora et al., 2020); parkinsonism (Nora et al., 2020); bronchial asthma (Nora et al., 2020), cardiovascular disorder (Nora et al., 2020)	conjunctival congestion (66.6) ocular burning sensation (8.33), watering (8.33), hordeolum externum (8.33)	Within 3 weeks of COVID symptoms (50 %) Before COVID symptoms (41.6 %)	N/A	N/A	N/A
Stephan et al. (2021)	France	20	N/A	Unilateral corneal injury (Qu et al., 2021), Superficial punctate keratitis without ulcer (1 8 0), Bilateral subconjunctival hemorrhages (Chen and Jungang, 2020)	N/A	Vit A ointment (for corneal injury)	Fully resolved	Prone to eye damage because of sedation, no blinking, and poor eyelid occlusions are prone to eye damage
Meduri et al. (2020)	Italy	29 patients	Hypertension, (86.2), Diabetes (62.1), Obesity (44.8), Coronary heart disease (27.5), Cerebrovascular disease, (24.1), Atrial fibrillation (24.1), Neurological disease (13.8), Cancer (Oren et al., 2021), BPCO (Oren et al., 2021), Chronic renal disease, (24.1)	Hyperemia (24.1), Chemosis (3.4), Secretion (6.9), Lid margin hyperemia (34.5), Crusted eyelashes (24.1), Meibomian orifices abnormalities (20.7)	N/A	N/A	Tear analysis did not reveal the presence of SARS-CoV-2. Ocular symptoms are common in patients with COVID-19	N/A
Oncul et al. (2021)	Turkey	359	N/A	Various ocular diseases (4.5) conjunctivitis	N/A	N/A	N/A	N/A
Oren et al. (2021)	Turkey	Total 60 COVID-19 group 35 Control group 25	N/A	Macular and peripapillary retinal nerve fiber layer (RNFL) thickness measurements, each retinal layer thickness of all participants was done 14–30 days after COVID-19 symptom onset	N/A	N/A	The mean value of central macular thickness and the mean values of the ganglion cell layer and inner nuclear layer thickness was significantly higher in the COVID-19 group	N/A
Oren and Kocabas (2022)	Turkey	COVID-19 group 34 Control group 34	N/A	Corneal endothelial cell morphology, increase in CV value	N/A	N/A	N/A	N/A
Perez-Chimal et al. (2021)	USA	15 newborns	N/A	Periorbital edema (1 0 0), Hemorrhagic conjunctivitis (73.3), Corneal edema (Ma et al., 2020), Hyaline secretion (1 0 0), Rubeosis (6.6)	N/A	N/A	N/A	N/A
Pirraglia et al. (2020)	Italy	43	N/A	No further retinal manifestation related to COVID-19 infection was found in our cohort	ophthalmological screening was performed after a median of 21.5 days	N/A	N/A	N/A
Ranzenigo et al. (2021)	Italy	53 patient	N/A	Conjunctivitis symptoms 37 Physician-assessed ocular signs 28	N/A	N/A	N/A	Plasma levels of Interleukin-6 were higher in patients with signs or symptoms in comparison with those without them: 43.5 pg/ml (19.7–49.4) vs. 8 pg/ ml (3.6–20.7) Red cell distribution width was also significantly higher
Riotto et al. (2022)	Switzerland	172 patient	Arterial hypertension, or Dyslipidemia (Sen et al., 2021); Hypertension (10.5), Coronary disease (5.3), Diabetes (36.8)	Cotton wool spots (CWS) and/or hemorrhages (Seah and Agrawal, 2020)	N/A	All subjects received 6 mg of dexamethasone daily since their admission	All patients were symptom-free 3 months after screening.	Diabetes history, overweight, and elevated C-reactive protein were more frequently observed among patients with retinal abnormalities, while a history of systemic hypertension was more frequently observed among patients without retinal findings
Rodriguez-Ares et al. (2021)	Spain	56 patients	Hypertension (48.2), diabetes (28.6), cancer (23.2), heart disease (21.4), obesity (17.9), chronic lung disease (Khan et al., 2021)	Grittiness (16.1), ocular pain (7.1), photophobia (1.8), blurry vision (3.6), conjunctival hyperemia (3.6), itching (3.6), secretion (7.1)	Average of 7.1 days (range 1–20) before ocular testing	N/A	No association was found between positive ocular samples and ocular symptoms	N/A.
Rokohl et al. (2020)	Germany	108 patients	N/A	burning sensations (36.1), epiphora (34.3), redness (25.9)	1.96 ± 3.17 days after the beginning of COVID-19	N/A	Do not need treatment	N/A
Kumar et al. (2021)	India	2742	N/A	Bilateral viral conjunctivitis, orbital cellulitis secondary to pansinusitis	Ocular examinations were performed every 72 h	N/A	N/A	N/A
Ma et al. (2020)	China	216 pediatric patients	N/A	Exhibited conjunctival discharge (12.5), eye rubbing (8.8), conjunctival congestion (2.3)	N/A	Observation without treatment and minimal eye drop	Complete recovery for 41 patients and persistent eye rubbing for the other 8.	Children with systematic symptoms had a higher chance of developing ocular manifestations
Jiang et al. (2021)	China	255 COVID-19	N/A	Asthenopia (4.3), mild conjunctival congestion, and serous secretion (0.8)				
Hepokur et al. (2023)	Turkey	16 COVID-19 17 control	N/A	Decrease in Subfoveal choroidal thickness (SFC), SFCT increase in late post-infectious period, peripapillary choroidal thickness, Decrease in choroidal stroma and blood vessels	N/A	N/A	N/A	N/A
Zhou et al. (2020)	China	243 patients	N/A	Exhibited ocular manifestations (6.6)	N/A	N/A	N/A	N/A
Wang et al. (2021)	China	42 patients	N/A	Dry eye disease (61.9)	N/A	N/A	N/A	N/A
Dag Seker and Erbahceci Timur (2021)	Turkey	32 COVID-19 36 control	N/A	Thinner macular retinal nerve fiber layer (RNFL) of inner and outer nasal and outer inferior quadrants	Eye examination was performed after 60.5 ± 38.6 days after COVID-19 confirmation	N/A	N/A	Patients with ocular pain had Thinner Superonasal and inferotemporal sectors of the Peripapillary retinal nerve fiber layer
Layikh et al. (2021)	Iraq	186	N/A	Conjunctivitis (13.4)	N/A	No treatment	All symptoms disappear within a few days	No significant association between gender and conjunctivitis prevalence, significant association between conjunctivitis and severity of the disease
Sehgal et al. (2021)	India	804 COVID-19	Diabetes mellitus, hypertension, obesity (81.5)	Conjunctival hyperemia (Jin et al., 2020); follicular reaction in palpebral conjunctiva (65.6), chemosis (Bypareddy et al., 2021)	4.52 ± 1.47 days from systemic manifestation to the onset of ocular manifestations	N/A	N/A	N/A
Reinhold et al. (2021)	Switzerland	10 COVID-19 5 control	N/A	swollen endothelial cells in congested choroidal vessels (Ma et al., 2020)	N/A	N/A	N/A	N/A
Jidigam et al. (2022)	USA	7 COVID-19 6 control group	N/A	Hemorrhagic spots and increased vitreous, increased retinal thickness, changes in retinal microvasculature, increased inflammation, gliosis, localized density changes, and increased inflammation	N/A	N/A	N/A	N/A
Wan et al. (2022)	China	228 COVID-19 109 Control	N/A	Meibomian gland dysfunction (MGD), ocular surface staining score, shorter Tear Break-up time in patients requiring supplementary oxygen during hospitalization	1 new ocular manifestation, 1 week before the COVID-19 diagnosis (Maychuk et al., 2020), ocular symptoms 4 weeks following diagnosis of COVID-19 (21.5) Evaluation was done within 52.23 ± 16.12 days after their COVID-19 positive test	N/A	N/A	N/A
Bayram et al. (2022)	Turkey-USA	53 COVID-19 group 53 control group	N/A	Outer plexiform layer thickness, choroidal thickness, low choroidal vascularity, increase in the stromal area to vascular area Significant increase in all quadrants of the peripapillary retinal nerve fiber layer (RNFL) thickness, significantly higher reflectivity of OCT echo of the choroid, and peripapillary RNFL	Day of patient hospitalization and third month of follow-up after recovery	N/A	N/A	Enhance in the outer plexiform layer thickness, mean choroidal thickness, the stromal area to vascular area (S/V) ratio of the choroid, peripapillary retinal nerve fiber layer (RNFL) thicknesses, and The reflectivity of OCT echo of the was observed in patients compared to the control group while a decrease in choroidal vascularity was detected.
Abdelkader et al. (2021)	Egypt	9	renal failure on hemodialysis (11.1)	All patients: edema and erythema of eyelids, severe conjunctival and ciliary injection, subconjunctival hemorrhage, corneal edema and infiltration, dense inflammatory coagulum in the anterior chamber, axial proptosis, limitation of the ocular motility. Ocular B-scan ultrasonography: medium to highly reflective floaters and membranous echoes with loculated opacities in the vitreous cavity more condensed posteriorly with choroidal thickening and the retina was in place Orbital CT: mild proptosis, haziness of orbital fat, in all cases. Mucoperiosteal thickening (11.1)	N/A	IV vancomycin, ceftazidime with oral metronidazole, Topical moxifloxacin hydrochloride 0.5 %, a topical combination of dexamethasone and tobramycin, and cycloplegic, Vitreous tap with intravitreal injection of vancomycin (1 mg/0.1 ml) and ceftazidime (2.25/0.1 ml)	3 patients died, atrophia bulbs in 4 eyes and preserved eyeball with complete visual loss in 2 patients	N/A
Abrishami et al. (2020)	Iran	142	Cataract (7.7), diabetic retinopathy (6.3)	Tearing (23.2), red eyes (Oren et al., 2021); eye irritation (13.4), eye itching (8.5), foreign body sensation (2.8), periorbital pain (3.5), photophobia (0.7), blurred vision (0.7), conjunctival swelling (15.5), conjunctival hyperemia (28.9), Chemosis (15.5)	N/A	N/A	N/A	The percentage of patients with ≥ 1 ocular manifestations was significantly higher in those admitted to ICU compared to the non-ICU group. Among all patients, the most common finding was conjunctival hyperemia. Among ICU patients, the most common finding was chemosis.
Akturk Acar et al. (2022)	Turkey	15	N/A	Bilateral conjunctivitis (26.7), an avascular area in Zone-III (Han et al., 2020)	The first: During hospitalization following negative RT-PCR result The second: 1 month later	Supportive treatments	N/A	N/A
Ahuja et al. (2020)	USA	1	N/A	Left eye irritation, upper eyelid swelling, erythematous, swollen and had to crust along the lashes, mild inflammation, and injection of the conjunctiva	Past 24 h	Doxycycline 100 mg to use if the symptoms worsened or did not improve	Clinically improved within six days of his initial presentation	N/A
Sezgin Akcay et al. (2021)	Turkey	1083	HTN (3 %), DM (1.3), allergic asthma (0.2 %), CHF (0.9 %), Hashimoto disease (0.3 %), CRF (0.2 %), RA (0.1), and lymphoma (0.1 %)	Sore eye or burning sensation (Jin et al., 2020), foreign body sensation ± burning sensation (3.6), red eye ± foreign body sensation, burning sensation, pain, itching (3.4)	Conjunctivitis symptoms manifested at the first and second week of disease onset in (28.5) and patients (71.4), respectively	N/A	N/A	The inpatient group had higher rates of comorbidity, ophthalmic medication, chronic ocular disease, and previous ocular surgery, but not contact lens wear compared to the outpatient group.
Boz et al. (2021)	Turkey	50	HTN (Maychuk et al., 2020), COPD (Wiersinga et al., 2020), Asthma (Wiersinga et al., 2020), DM (Khalili et al., 2020)	Follicular conjunctivitis (Baig, 2020); Blepharoconjunctivitis (Renu et al., 2020), Blepharitis (Renu et al., 2020), Papillary conjunctivitis (7.5), Anterior uveitis (Wiersinga et al., 2020); Presence of cataract (Wiersinga et al., 2020), Presence of pterygium (Khalili et al., 2020)	Within 2 weeks after COVID-19 infection had been confirmed	Symptomatic treatment (preservative-free artificial tears, cold compress, and lubricating ophthalmic ointment)	All resolved	N/A
Bypareddy et al. (2021)	India	138	N/A	A single streak of superficial retinal hemorrhage at the posterior pole of the fundus in the left eye of one patient (0.72)	6 days from the infection symptoms onset	N/A	N/A	No lesions that can be attributed to COVID-19 were found in those with mild to moderate COVID-19 symptoms
Chen et al. (2020)	China	535	HTN (Chen et al., 2020), Hyperlipidaemia (1.1), DM (7.1), Cardiovascular and cerebrovascular diseases (3.3), Respiratory system disease (6.9), Hematological system disease (0.5), CKD (0.5), Chronic liver disease (4.7), Autoimmune disease (1.9)	*Without conjunctival congestion group:* Conjunctival secretion (8.7), Ocular pain (3.5), Foreign body sensation (11.4), Photophobia (2.6), Blurred vision (12.8), Dry eye (20.1), Tearing (9.6), Itching (9.6), *With conjunctival congestion group:* Conjunctival secretion (29.6), Ocular pain (18.5), Foreign body sensation (18.5), Photophobia (11.1), Blurred vision (11.1), Dry eye (Rodriguez-Ares et al., 2021), Tearing (22.2), Itching (14.8)	Conjunctival congestion 0–3 days in 7 patients, 4–7 days in 1 patient, 8–14 days in 6 patients, and 15–28 days in 6 patients after clinical symptoms	N/A	N/A	Conjunctival congestion lasted for an average of 5.9 ± 4.5 days. A significant association between hand–eye contact and conjunctival congestion appeared.
Scalinci and Trovato (2020)	Italy	5	N/A	chemosis, epiphora, photophobia, Conjunctivitis	Initial presentation in all patients	Moxifloxacin eye drops four times a day for 5 more days.	Resolved	Conjunctivitis was the only sign and symptom of COVID-19 infection.
Gangaputra and Patel (2020)	USA	144 COVID-19 positive 306 COVID-19 negative	N/A	Eye pain 19.4, Photophobia 13.9, flashes/floaters 11.8, blurry vision 11.1, red eyes 10.4	1 to 4 weeks following the results of 69 their COVID-19 testing	N/A	26.5 % of patients were suffering from ocular symptoms despite recovery from systemic infection	Red eyes and epiphora were more likely to be found in COVID-19-negative patients relative to COVID-19 positive ones.

### 2.4 Quality assessment and bias risk evaluation

As mentioned above, to enhance the reliability and soundness of our research, we employed the items of PRISMA checklist in this review study. Moreover, we assessed the risk of bias in the included studies using the Newcastle-Ottawa Scale (NOS). The criteria of this tool, which include selection, comparability, and exposure/outcome, have respective values of 4, 2, and 3 for individual studies, as shown in Table 1. The sum of these values gives a maximum possible score of nine for each included study.

## 3 Results

**Fig. 1:**
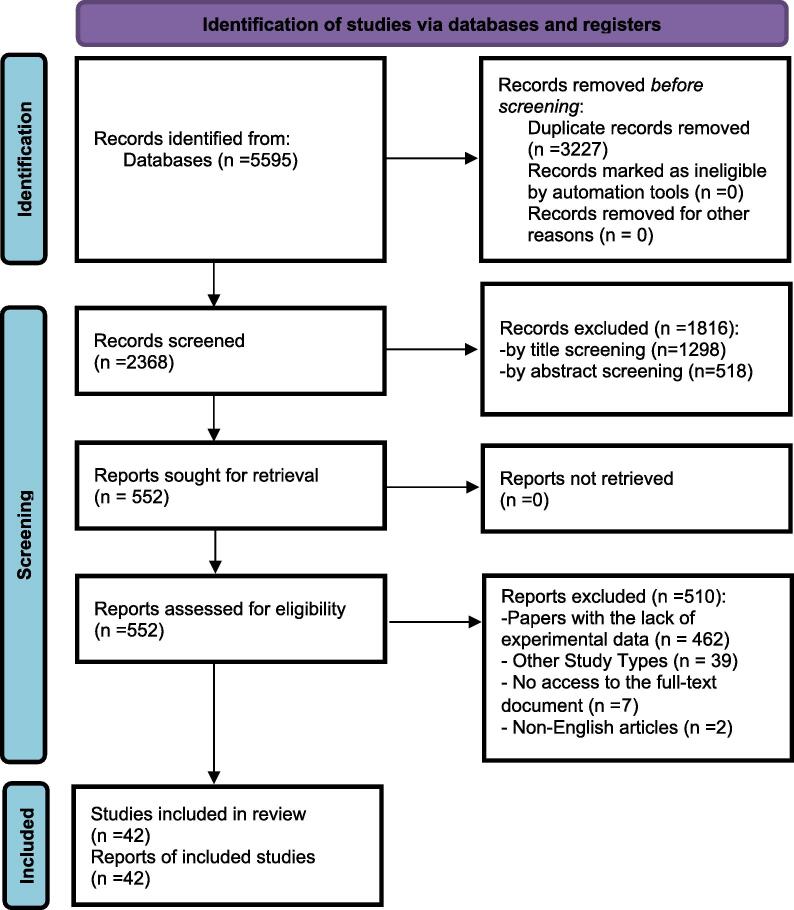
PRISMA 2020 flow diagram of study retrieval process.

In this review, we investigated the ocular manifestations in COVID-19 patients by examining available evidence. We initially identified 5,595 articles. After eliminating 3,227 duplicates, we screened 2,368 articles based on their titles and abstracts. In the first phase of screening, 1,816 articles were excluded based on our inclusion and exclusion criteria. During the second phase of screening, 552 articles were reviewed, from which we extracted data from 42 articles. The selection process is illustrated in the PRISMA diagram (Fig. 1). Most of the studies were cross-sectional (n = 33), followed by case-control studies (n = 3), cohort studies (n = 3), case-report (n = 2), and case-series (n = 1). Most of studies have been conducted in Turkey (n = 10); followed by China (n = 7), Italy (n = 4), India (n = 6), the USA (n = 4), with two studies each from Brazil, and Switzerland. Single studies were conducted in France, Spain, Germany, Iraq, Egypt, Iran, and a multi-country study was undertaken in both Turkey and the USA.

Comorbidities were reported as follow: hypertension in 14 articles, Diabetes Mellitus in 12 articles, obesity and cerebrovascular in 4 articles, cancer and asthma in 3 articles; and cardiovascular, cataract, renal disease, respiratory system disease, and dyslipidemia were each mentioned in 2 articles. Autoimmune disease, hyperlipidemia, chronic liver disease, CKD, COPD, Hashimoto disease, diabetic retinopathy, CRF, glaucoma, nephritis, thyroid disorders, and neurological disorders were each discussed in one article. Ocular manifestations were reported in the majority of the studies and were significantly more prevalent in COVID-19 patients compared to control groups. The most common ocular manifestation was conjunctivitis, mentioned in 24 articles, followed by photophobia, burning, chemosis, itching, and ocular pain in fourteen, seven, seven, six, and five articles, respectively. Less prevalent manifestations were as follows: tearing, red eyes, irritation, eye pain, blurry vision, and retinal hemorrhage. Some studies reported treatments for these manifestations, with most patients recovering fully (Giampietro et al., 2023, Silveira et al., 2022, Pirraglia et al., 2020, Wang et al., 2021, Reinhold et al., 2021, Wan et al., 2022, Abrishami et al., 2020). However, one study noted visual loss in two patients (Chen et al., 2020).

Based on a cross-sectional study of 104 COVID-19 patients of which 36 exhibited ocular symptoms, it was observed that ocular manifestations related to COVID-19 can manifest at various stages. Among those with ocular symptoms, 77.7 % developed them within three weeks of the onset of COVID-19 symptoms. Specifically, 50 % exhibited ocular symptoms concurrently with general symptoms. Additionally, 41.6 % showed ocular symptoms before the onset of COVID-19 symptoms, with 11.11 % presenting them one day before flu-like symptoms and another 11.11 % displaying them three days afterward (Silveira et al., 2022).

The interval between the ocular testing and the ophthalmological screening varied, with a median of 21.5 days (Pirraglia et al., 2020, Wan et al., 2022), an average of 7.1 days (range 1–20 days) (Rodriguez-Ares et al., 2021), and within 52.23 ± 16.12 days following a positive COVID-19 test (Wan et al., 2022). In some cases, ocular tests were performed every 72 h (Kumar et al., 2021).

Conjunctivitis symptoms appeared as particular ocular manifestations at various times, with 28.5 % of patients exhibiting them during the first week of disease onset and 71.4 % exhibiting them during the second. Some individuals developed conjunctival congestion 0–3 days after developing clinical symptoms, while others developed it 15–28 days after developing clinical symptoms (Jidigam et al., 2022). Overall, the timing and presentation of ocular symptoms in COVID-19 patients varied, indicating that ocular involvement can happen at various times over the course of the illness (Giampietro et al., 2023, Sharma et al., 2022, Oren and Kocabas, 2022, Riotto et al., 2022, Dag Seker and Erbahceci Timur, 2021).

According to the findings, conjunctivitis is a rare and self-limited consequence in adults with COVID-19. However, the presence of coronavirus receptors on the surface of the human eye and the lacrimal glands of mice raises concerns about SARS-CoV-2 infection. Individuals with ocular pain had thinner peripapillary retinal nerve fiber layers, and those with fever and ocular involvement typically had greater levels of CRP, neutrophil counts, and ESR, while having lower levels of lymphocytes. Also, the inpatient group of patients had a higher prevalence of ocular symptoms, comorbidities, chronic ocular disease, and prior ocular surgery. Also, patients with signs or symptoms had greater plasma levels of interleukin-6 and red cell distribution width, and patients with retinal abnormalities were more likely to have a history of diabetes (Baig, 2020, Firat and Kobat, 2021, Ganesh and Mohanan-Earatt, 2022, Shaikh et al., 2022, Shen et al., 2021, Sindhuja et al., 2020, Pirraglia et al., 2020, Ranzenigo et al., 2021, Dag Seker and Erbahceci Timur, 2021, Jidigam et al., 2022, Wan et al., 2022, Abdelkader et al., 2021), be overweight (Baig, 2020, Sindhuja et al., 2020, Ranzenigo et al., 2021, Dag Seker and Erbahceci Timur, 2021), or have raised C-reactive protein levels (Pirraglia et al., 2020).

## 4 Discussion

### 4.1 Overview of findings

Evidence indicates that COVID-19 exhibited a wide range of signs and symptoms. Although ocular manifestations in COVID-19 patients are typically infrequent, our systematic review aimed to evaluate these symptoms, potentially aiding in the disease's earlier diagnosis. In this systematic review, the authors encompassed 42 studies, with conjunctivitis emerging as the most frequently reported ocular symptom (n = 24 papers), followed by photophobia (n = 14), burning (n = 7), chemosis (n = 7), itching (n = 6), and ocular pain (n = 5). Some less common manifestations were as follows: tearing, red eyes, irritation, eye pain, blurry vision, and retinal hemorrhage.

### 4.2 Ocular manifestations

A *meta*-analysis involving 8,219 COVID-19 patients indicated an 11.03 % prevalence of ocular symptoms. It highlighted that foreign body sensation or dry eye was the most prevalent ocular symptom (16.0 %), followed by eye redness (13.3 %) and tearing (12.8 %). Similar to our findings, Nasiri et al. also reported that the most prevalent ocular disease was conjunctivitis (88.8 %) (Nasiri et al., 2021). Another *meta*-analysis among 5,717 COVID-19 patients reported that the most frequent ocular symptoms were; hyperemia of conjunctiva, conjunctival discharge, tearing and foreign body sensation. This study also emphasized that severe COVID-19 patients were 2–3 times more likely to be have ocular manifestations than mild cases (Zhong et al., 2021). Soltani et al. in their systematic review of 3,650 COVID-19 demonstrated that the prevalence of having at least one type of ocular manifestations was 23.77 %. The most frequent symptom was dry eyes (13.66 %), followed by conjunctival hyperemia (13.41 %), conjunctival congestion/conjunctivitis (9.14 %), ocular pain (10.34 %,), irritation/itching/burning sensation (9.34 %,), and foreign body sensation (5.24 %) (Soltani et al., 2022).

In our study, conjunctivitis emerged as the most frequently observed ocular symptom, reported in 24 articles. Aligned with our findings, N. Shaikh et al. (Shaikh et al., 2022), reported that 33.3 % of COVID-19 patients exhibited conjunctivitis, making it the predominant ocular manifestation, followed by eye pain at 23.1 %. Another study showed that 13.4 % of patients experienced conjunctivitis, which significantly correlated with disease severity (Shen et al., 2021). The underlying pathogenic pathways of conjunctival COVID-19 infection remain unclear. The ocular surface might serve as an entry point for the virus, either through hand-to-eye contact or exposure to aerosolized droplets. Two critical components facilitating the virus's entry into host cells are the cell surface protease enzyme (TMPRSS2) and the ACE-2 receptor. The presence of these receptors on the ocular surface is a subject of debate. However, research utilizing immunohistochemistry analysis has indicated the ACE-2 receptor's distinct presence in the conjunctiva, cornea, and limbus. Additionally, conjunctival samples have shown expression of TMPRSS2 (Zhou et al., 2020). The research showing that SARS-CoV-2 RNA may be found in the conjunctival mucosa and tears of COVID-19 patients reinforces the idea that the eye may be a potential site of infection (Mohammad Alrawashdeh et al., 2021). SARS-CoV-2 is thought to spread via the conjunctival sac tears and nasolacrimal ducts and finally proceed to the respiratory system (Qing et al., 2020). Additionally, it has been suggested that the direct viral assault from the eyes could be maintained by a strong immune response that may result in significant tissue injury. As a result, autoimmune and auto-inflammatory responses may both be at play (Gulati et al., 2020). The symptoms of conjunctivitis associated with COVID-19 are comparable to those of other viral types. Most often, the patients have chemosis, epiphora, follicular response of the tarsal conjunctiva, minor eyelid edema, watery discharge, and swollen submaxillary and preauricular lymph nodes (Bertoli et al., 2020). The COVID-19 conjunctival symptoms seem to be self-limiting. Some patients have shown symptom relief after the application of topical treatments like ganciclovir and ribavirin (Sen et al., 2021).

Regarding ocular chemosis, a notable symptom observed in various studies, G. Sehgal et al. examined 309 COVID-19 patients with ocular complications and found that 58.2 % exhibited chemosis (Sehgal et al., 2021). Another study revealed that 15.5 % out of 142 COVID-19 patients presented chemosis as an ocular symptom (Abrishami et al., 2020). Factors such as decreased venous return and elevated hydrostatic pressure resulting from positive pressure ventilation or tight endotracheal tube tape, mainly due to prolonged recumbency, are potential risk factors for conjunctival chemosis (Sen et al., 2021). Some other manifestations such as photophobia, ocular pain, itching, and burning have also been reported as ocular manifestations of COVID-19 patients (Shaikh et al., 2022, Rodriguez-Ares et al., 2021, Abrishami et al., 2020). Therefore, medical personnel in close contact with COVID-19 patients should wear face shield or eye protection glasses, given the virus's ability to adhere to the ocular surface and enter the respiratory system through the eye (Qing et al., 2020).

In addition to the direct effects of the virus on the eyes, its treatments and immunizations have similar impacts. Some medications for COVID-19, such as chloroquine and hydroxychloroquine—commonly used treatments—may pose risks to ocular health. Prolonged use of these drugs can lead to retinal toxicity, although short-term use seems safe. Interferon, another treatment option, has been linked to side effects including retinopathy, conjunctivitis, corneal ulcers, optic neuropathy, and epithelial abnormalities (Asili et al., 2021, Eissa et al., 2022). Ribavirin is also identified to have side effects such as serous retinal detachment, retinopathy, retinal vein occlusion, and ischemic optic neuropathy. Corticosteroids also have known ocular side effects in systemic treatment, which comprise glaucoma, cataracts, and central serous chorioretinopathy (Samaranayake et al., 2020).

In regards to protective measures and ocular transmission of COVID-19, Samaranayake et al. in their systematic review of 21 papers found that using layered, masks/respirators that fit on face, and protective-eyewear can curb COVID-19 transmission among healthcare workers. They also specified that a combination of intervention such as face mask and a face shield, can resist aerosol inhalation more effectively than either alone (Matos et al., 2021). Similarly, a review showed that SARS-CoV-2 can be transmitted effectively through the eyes which signifies the importance of eye-protection tools like goggles for health care workers or potential carriers of the virus (Chu et al., 2020). One study supported by the WHO for guidelines establishment on social distancing also proposed that eye protection can be effective in transmission prevention in the community setting and provides additional benefits (Davis et al., 2022). Another review also asserted that eyes are an additional entry route for SARS-CoV-2, and found that SARS-CoV-2 is most probable to be extracted from the ocular secretions of high-viral load patients that have conjunctivitis, as the ocular fluid can serve as a source of viral replication and transmission vector to corneal, conjunctival, and nasolacrimal tissue. Thus they also have signified the rule of protective eye-wear for prevention of COVID-19 transmission (Masiá et al., 2021).

### 4.3 Time of onset

In terms of ocular manifestation timeline, several studies have shown a correlation with the timing of the increase in immunoglobulin levels. In essence, high IgG concentrations, early seroconversion, and an elevated viral load have all been linked to severe COVID-19. It has been demonstrated that a mild infection results in delayed seroconversion and longer viral shedding. Due to the longer time being exposed to viral antigens, there is a delayed viral clearance and also increased serum levels of IgG at 6–8 weeks (Jin et al., 2020, Danthuluri and Grant, 2020). In an assessment of 27 COVID-19 patients, the onset of ocular symptoms spanned a broad timeline. For some, ocular manifestations were the initial symptom of the COVID-19 infection, while for others, these symptoms appeared anywhere from 0 to 28 days after the onset of clinical symptoms (Chen et al., 2020). Another study found that ophthalmological symptoms manifested between one and seven days following the appearance of clinical symptoms (Boz et al., 2021). The ocular symptoms may appear at any stage of the disease. Conjunctivitis, as a medical sign of COVID-19, usually appears at the beginning of the disease. If ocular pathology develops later in the clinical phase, it seems to be linked to COVID-19 being more severe. Although early detection of COVID-related ocular symptoms should limit the spread of the illness in general, evidence for direct transmission through eye mucosa is currently lacking (Danthuluri and Grant, 2020).

### 4.4 Outcome

Unfortunately, 26 out of 42 articles neither reported the outcome of patients nor did followed patients. However, the majority of studies that included a short follow-up reported that most of the ocular manifestations resolved spontaneously within few days or with supportive treatments. In cases of severe conditions, patients underwent medical treatments such as Vit-A/healing ointment, antibiotic eye drops, topical steroids, IV steroids, etc. and even surgery, leading to clinical improvement and complete recovery. While most symptoms disappeared and patients became symptom-free, some individuals continued to experience ocular discomfort, such as eye rubbing, for several months.

### 4.5 Limitation

The present study has some limitations. Firstly, the study design of included studies did not allow for reliable causal inferences. Additionally, there were few studies on some of the discussed matters, which may reduce the validity and reliability of reported outcomes. Nevertheless, this study may provide relevant insights for future research to conduct original studies and/or meta‐ analyses to precisely determine the association between COVID-19 and ocular manifestations. Furthermore, our recommendation for future studies is to continuously monitor patients in order to identify long-term effects of COVID-19 on eyes.

## 5 Conclusion

Although ocular manifestations of COVID-19 appear to be infrequent, they present a broad spectrum of symptoms, ranging in severity from mild eye redness or itching that may need no or only supportive treatment, to severe cases requiring medical intervention and possibly surgery. While there have been few reports of severe conditions resulting in visual loss, the majority of patients responded favorably to treatments, with full recoveries being attainable. These findings highlight the need for a comprehensive clinical assessment, encompassing not just the commonly recognized respiratory symptoms, but also potential ocular complications. Clinicians should be aware of the possibility of ocular symptoms as an early or concurrent presentation of COVID-19. Regular ophthalmic examinations, particularly for hospitalized patients, can aid in early detection and management and future policies should consider updating guidelines to include routine eye examinations for COVID-19 patients, particularly those exhibiting severe respiratory symptoms, as this could be indicative of systemic disease severity.

## 6 Future research

Few studies have investigated the ocular manifestations of COVID-19 among the children or the elderly, or the potential differences between sexes. In order to fill that gap in literature, the ocular symptoms among specific age or gender sub groups, more studies are needed. In addition, the exact mechanism of SARS-CoV-2 transmission via tear is unknown and uncertain thus further studies can be conducted to shed light on that part.

## CRediT authorship contribution statement

**SeyedAhmad SeyedAlinaghi:** Writing – original draft. **Esmaeil Mehraeen:** Writing – original draft, Investigation, Conceptualization. **Arian Afzalian:** Writing – original draft. **Mohsen Dashti:** Writing – original draft. **Afsaneh Ghasemzadeh:** Writing – original draft. **Ava Pashaei:** Writing – original draft. **Amir Masoud Afsahi:** Writing – original draft. **Seyed Saeed Tamehri Zadeh:** Writing – original draft. **Iman Amiri Fard:** Writing – original draft. **AmirMohammad Vafaee:** Writing – original draft. **Ayoob Molla:** Methodology, Investigation. **Ramin Shahidi:** Writing – original draft. **Ali Dadjou:** Writing – original draft. **Mohammad Amin Habibi:** Writing – original draft. **Pegah Mirzapour:** Writing – original draft, Supervision. **Omid Dadras:** Writing – review & editing.

## Declaration of competing interest

The authors declare that they have no known competing financial interests or personal relationships that could have appeared to influence the work reported in this paper.

## Data Availability

The data that has been used is confidential.

## References

[b0005] Abdelkader M., Elshafei A.M.K., Nassar M.M., Abu Elela M.A., Abdallah R.M.A. (2021). Combined endophthalmitis and orbital cellulitis in patients with corona virus disease (COVID-19). J. Ophthalmic. Inflamm. Infect..

[b0010] Abrishami M., Tohidinezhad F., Daneshvar R., Omidtabrizi A., Amini M., Sedaghat A. (2020). Ocular Manifestations of Hospitalized Patients with COVID-19 in Northeast of Iran. Ocul. Immunol. Inflamm..

[b0015] Ahuja A.S., Farford B.A., Forouhi M., Abdin R., Salinas M. (2020). The Ocular Manifestations of COVID-19 Through Conjunctivitis. Cureus..

[b0020] Akturk Acar F., Esenulku M.C., Hekimoglu B. (2022). Retinal Findings of Hospitalized Neonates Recovered from COVID-19 Infection: A Prospective, Observational, Descriptive Study. J. Trop. Pediatr..

[b0025] Al-Namaeh M. (2021). COVID-19 and conjunctivitis: a meta-analysis. Therapeutic Adv. Ophthalmol..

[b0030] Asili P., Mirahmad M., Tabatabaei-Malazy O., Manayi A., Haghighat E., Mahdavi M. (2021). Characteristics of published/registered clinical trials on COVID-19 treatment: A systematic review. Daru.

[b0035] Baig AM. Chronic COVID syndrome: Need for an appropriate medical terminology for long-COVID and COVID long-haulers. 2020. 10.1002/jmv.26624 33095459

[b0040] Bayram N., Gundogan M., Ozsaygili C., Adelman R.A. (2022). Posterior ocular structural and vascular alterations in severe COVID-19 patients. Graefes Arch. Clin. Exp. Ophthalmol..

[b0045] Bedford J., Enria D., Giesecke J., Heymann D.L., Ihekweazu C., Kobinger G. (2020). COVID-19: towards controlling of a pandemic. Lancet.

[b0050] Bertoli F., Veritti D., Danese C., Samassa F., Sarao V., Rassu N. (2020). Ocular Findings in COVID-19 Patients: A Review of Direct Manifestations and Indirect Effects on the Eye. J. Ophthalmol..

[b0055] Boz A.A.E., Atum M., Cakir B., Karabay O., Celik E., Alagoz G. (2021). Outcomes of the Ophthalmic Examinations in Patients Infected by SARS-CoV-2. Ocul. Immunol. Inflamm..

[b0060] Bypareddy R., Rathod B.L.S., Shilpa Y.D., Hithashree H.R., Nagaraj K.B., Hemalatha B.C. (2021). Fundus evaluation in COVID-19 positives with non-severe disease. Indian J. Ophthalmol..

[b0065] Chen L., Deng C., Chen X., Zhang X., Chen B., Yu H. (2020). Ocular manifestations and clinical characteristics of 535 cases of COVID-19 in Wuhan, China: a cross-sectional study. Acta Ophthalmol..

[b0070] Chen L., Jungang X. (2020). Interpretation of “the diagnosis and treatment plan for COVID-19 (the seventh trial edition)”. Her Med..

[b0075] Chen L., Liu M., Zhang Z., Qiao K., Huang T., Chen M. (2020). Ocular manifestations of a hospitalised patient with confirmed 2019 novel coronavirus disease. Br. J. Ophthalmol..

[b0080] Chu D.K., Akl E.A., Duda S., Solo K., Yaacoub S., Schünemann H.J. (2020). Physical distancing, face masks, and eye protection to prevent person-to-person transmission of SARS-CoV-2 and COVID-19: a systematic review and meta-analysis. Lancet.

[b0085] Dag Seker E., Erbahceci Timur I.E. (2021). COVID-19: more than a respiratory virus, an optical coherence tomography study. Int. Ophthalmol..

[b0090] Danthuluri V., Grant M.B. (2020). Update and Recommendations for Ocular Manifestations of COVID-19 in Adults and Children: A Narrative Review. Ophthalmol Ther..

[b0095] Davis G., Li K., Thankam F.G., Wilson D.R., Agrawal D.K. (2022). Ocular transmissibility of COVID-19: possibilities and perspectives. Mol. Cell. Biochem..

[b0100] Domínguez-Varela I.A., Rodríguez-Gutiérrez L.A., Morales-Mancillas N.R., Barrera-Sánchez M., Macias-Rodriguez Y., Valdez-García J.E. (2021). COVID-19 and the eye: a review. Infect. Dis..

[b0105] Eissa M., Abdelrazek N.A., Saady M. (2022). Covid-19 and its relation to the human eye: transmission, infection, and ocular manifestations. Graefes Arch. Clin. Exp. Ophthalmol..

[b0110] Firat M., Kobat S. (2021). How are central foveal and choroidal thickness affected in patients with mild COVID-19 infection?. Bosn. J. Basic Med. Sci..

[b0115] Ganesh S.K., Mohanan-Earatt A. (2022). An analysis of the clinical profile of patients with uveitis following COVID-19 infection. Indian J. Ophthalmol..

[b0120] Gangaputra S.S., Patel S.N. (2020). Ocular Symptoms among Nonhospitalized Patients Who Underwent COVID-19 Testing. Ophthalmology.

[b0125] Giampietro B.V., Dutra S., Oliveira R.V.C., Biancardi A.L., Veloso V., Curi A.L.L. (2023). Ophthalmological Findings in Patients with SARS-CoV-2 Infection Examined at the National Institute of Infectious Diseases - INI/Fiocruz. Ocul. Immunol. Inflamm..

[b0130] Gulati A., Pomeranz C., Qamar Z., Thomas S., Frisch D., George G. (2020). A Comprehensive Review of Manifestations of Novel Coronaviruses in the Context of Deadly COVID-19 Global Pandemic. Am. J. Med. Sci..

[b0135] Han C., Duan C., Zhang S., Spiegel B., Shi H., Wang W. (2020). Digestive symptoms in COVID-19 patients with mild disease severity: clinical presentation, stool viral RNA testing, and outcomes. Am. J. Gastroenterol..

[b0140] Hepokur M., Gunes M., Durmus E., Aykut V., Esen F., Oguz H. (2023). Long-term follow-up of choroidal changes following COVID-19 infection: analysis of choroidal thickness and choroidal vascularity index. Can. J. Ophthalmol..

[b0145] Hutama S.A., Alkaff F.F., Intan R.E., Maharani C.D., Indriaswati L., Zuhria I. (2022). Recurrent keratoconjunctivitis as the sole manifestation of COVID-19 infection: a case report. Eur. J. Ophthalmol..

[b0150] Jiang B., Li S.J., Wang W.L., Hu M., He S., Cao J. (2021). Ocular manifestations and SARS-CoV-2 detection in tears and conjunctival scrape from non-severe COVID-19 patients. Int. J. Ophthalmol..

[b0155] Jidigam V.K., Singh R., Batoki J.C., Milliner C., Sawant O.B., Bonilha V.L. (2022). Histopathological assessments reveal retinal vascular changes, inflammation, and gliosis in patients with lethal COVID-19. Graefes Arch. Clin. Exp. Ophthalmol..

[b0160] Jin X., Lian J.-S., Hu J.-H., Gao J., Zheng L., Zhang Y.-M. (2020). Epidemiological, clinical and virological characteristics of 74 cases of coronavirus-infected disease 2019 (COVID-19) with gastrointestinal symptoms. Gut.

[b0165] Jin C.C., Zhu L., Gao C., Zhang S. (2020). Correlation between viral RNA shedding and serum antibodies in individuals with coronavirus disease 2019. Clin. Microbiol. Infect..

[b0170] Khalili M., Karamouzian M., Nasiri N., Javadi S., Mirzazadeh A., Sharifi H. (2020). Epidemiological characteristics of COVID-19: a systematic review and meta-analysis. Epidemiol. Infect..

[b0175] Khan S.I., Versha F., Bai P., Bachani P., Nawaz M.U., Kumar L. (2021). Frequency of ophthalmological findings in hospitalized COVID-19 patients. Cureus..

[b0180] Kumar K.K., Sampritha U.C., Prakash A.A., Adappa K., Chandraprabha S., Neeraja T.G. (2021). Ophthalmic manifestations in the COVID-19 clinical spectrum. Indian J. Ophthalmol..

[b0185] Layikh H.A., Hashim Z.A., Kadum A.A. (2021). Conjunctivitis and other ocular findings in patients with COVID-19 infection. Ann. Saudi Med..

[b0190] Ma N., Li P., Wang X., Yu Y., Tan X., Chen P. (2020). Ocular Manifestations and Clinical Characteristics of Children With Laboratory-Confirmed COVID-19 in Wuhan, China. JAMA Ophthalmol..

[b0195] Mahayana I.T., Angsana N.C., Kamila A., Fatiha N.N., Sunjaya D.Z., Andajana W. (2020). Literature review of conjunctivitis, conjunctival swab and chloroquine effect in the eyes: a current updates on COVID-19 and ophthalmology. J. Med Sci. (berkala Ilmu Kedokteran).

[b0200] Masiá M., Telenti G., Fernández M., García J.A., Agulló V., Padilla S. (2021). SARS-CoV-2 Seroconversion and Viral Clearance in Patients Hospitalized With COVID-19: Viral Load Predicts Antibody Response. Open Forum. Infect. Dis..

[b0205] Matos A.G., Sarquis I.C., Santos A.A.N., Cabral L.P. (2021). COVID-19: risk of ocular transmission in health care professionals. Rev. Bras Med. Trab..

[b0210] Maychuk D.Y., Atlas S., Loshkareva A. (2020). Ocular manifestations of coronavirus infection COVID-19 (clinical observation). Vestn. Oftalmol..

[b0215] Meduri A., Oliverio G.W., Mancuso G., Giuffrida A., Guarneri C., Venanzi Rullo E. (2020). Ocular surface manifestation of COVID-19 and tear film analysis. Sci. Rep..

[b0220] Mohammad Alrawashdeh H., Al Zubi K., Abdulmannan D.M., Al-Habahbeh O., Abu-Ismail L. (2021). Conjunctivitis as the only sign and symptom of COVID-19: A case report and review of literature. Qatar Med. J..

[b0225] Nasiri N., Sharifi H., Bazrafshan A., Noori A., Karamouzian M., Sharifi A. (2021). Ocular Manifestations of COVID-19: A Systematic Review and Meta-analysis. J. Ophthalmic Vis. Res..

[b0230] Nora R.L.D., Putera I., Khalisha D.F., Septiana I., Ridwan A.S., Sitompul R. (2020). Are eyes the windows to COVID-19? Systematic review and meta-analysis. BMJ Open Ophthalmology..

[b0235] Oncul H., Oncul F.Y., Alakus M.F., Caglayan M., Dag U. (2021). Ocular findings in patients with coronavirus disease 2019 (COVID-19) in an outbreak hospital. J. Med. Virol..

[b0240] Oren B., Aksoy Aydemir G., Aydemir E., Atesoglu H.I., Goker Y.S., Kiziltoprak H. (2021). Quantitative assessment of retinal changes in COVID-19 patients. Clin. Exp. Optom..

[b0245] Oren B., Kocabas D.O. (2022). Assessment of corneal endothelial cell morphology and anterior segment parameters in COVID-19. Ther. Adv. Ophthalmol..

[b0250] Perez-Chimal L.G., Cuevas G.G., Di-Luciano A., Chamartin P., Amadeo G., Martinez-Castellanos M.A. (2021). Ophthalmic manifestations associated with SARS-CoV-2 in newborn infants: a preliminary report. J. AAPOS.

[b0255] Pirraglia M.P., Ceccarelli G., Cerini A., Visioli G., d'Ettorre G., Mastroianni C.M. (2020). Retinal involvement and ocular findings in COVID-19 pneumonia patients. Sci. Rep..

[b0260] Qing H., Li Z., Yang Z., Shi M., Huang Z., Song J. (2020). The possibility of COVID-19 transmission from eye to nose. Acta Ophthalmol..

[b0265] Qu J.-Y., Xie H.-T., Zhang M.-C. (2021). Evidence of SARS-CoV-2 transmission through the ocular route. Clin. Ophthalmol..

[b0270] Ranzenigo M., Bruzzesi E., Galli L., Castagna A., Ferrari G. (2021). Symptoms and signs of conjunctivitis as predictors of disease course in COVID-19 syndrome. J. Ophthalmic Inflamm. Infect..

[b0275] Reinhold A., Tzankov A., Matter M.S., Mihic-Probst D., Scholl H.P.N., Meyer P. (2021). Ocular Pathology and Occasionally Detectable Intraocular Severe Acute Respiratory Syndrome Coronavirus-2 RNA in Five Fatal Coronavirus Disease-19 Cases. Ophthalmic Res..

[b0280] Renu K., Prasanna P.L., Gopalakrishnan A.V. (2020). Coronaviruses pathogenesis, comorbidities and multi-organ damage–A review. Life Sci..

[b0285] Riotto E., Megevand V., Megevand A., Marti C., Pugin J., Stangos A.N. (2022). Retinal Manifestations in Patients with COVID-19: A Prospective Cohort Study. J. Clin. Med..

[b0290] Rodriguez-Ares T., Lamas-Francis D., Trevino M., Navarro D., Cea M., Lopez-Valladares M.J. (2021). SARS-CoV-2 in Conjunctiva and Tears and Ocular Symptoms of Patients with COVID-19. Vision (basel).

[b0295] Rokohl A.C., Loreck N., Wawer Matos P.A., Zwingelberg S., Augustin M., Dewald F. (2020). More than loss of taste and smell: burning watering eyes in coronavirus disease 2019. Clin. Microbiol. Infect..

[b0300] Samaranayake L.P., Fakhruddin K.S., Ngo H.C., Chang J.W.W., Panduwawala C. (2020). The effectiveness and efficacy of respiratory protective equipment (RPE) in dentistry and other health care settings: a systematic review. Acta Odontol. Scand..

[b0305] Scalinci S.Z., Trovato B.E. (2020). Conjunctivitis can be the only presenting sign and symptom of COVID-19. Idcases..

[b0310] Seah I., Agrawal R. (2020). Can the coronavirus disease 2019 (COVID-19) affect the eyes? A review of coronaviruses and ocular implications in humans and animals. Ocul. Immunol. Inflamm..

[b0315] Sehgal G., Bal P., Bal B., Chopra R. (2021). Pattern of ocular manifestations and the prevalence of severe acute respiratory syndrome coronavirus-2 in tears of hospitalized coronavirus disease 2019 patients. Taiwan J. Ophthalmol..

[b0320] Sen M., Honavar S.G., Sharma N., Sachdev M.S. (2021). COVID-19 and Eye: A Review of Ophthalmic Manifestations of COVID-19. Indian J. Ophthalmol..

[b0325] Sezgin Akcay B.I., Kardes E., Kiray G., Ayaz B., Karakus Hacioglu G., Pala E. (2021). Evaluation of ocular symptoms in COVID-19 subjects in inpatient and outpatient settings. Int. Ophthalmol..

[b0330] Shaikh N., Al Mahdi H., Pai A., Pathare A., Abujaber A.A., Dsliva A. (2022). Ocular manifestations of COVID-19: facts and figures from a tertiary care center. Ann. Med..

[b0335] Sharma S., Yadav P., Shekhawat P., Lunia G., Taneja Y., Shekhawat K. (2022). Spectrum of Ocular Manifestations in SARS COV–2 Patients at Tertiary Care Center. Eur. J. Mol. Clinical Med..

[b0340] Shen J., Wu J., Yang Y., Wang P., Luo T., Guo Y. (2021). The paradoxical problem with COVID-19 ocular infection: Moderate clinical manifestation and potential infection risk. Comput. Struct. Biotechnol. J..

[b0345] Silveira A.K.T., Lynch M.I., Medeiros C.S.L., Moraes B.T., Remigio M.C., Paiva M.M.F. (2022). Ophthalmological findings in patients suspected with COVID-19 at a tertiary hospital in Pernambuco. Brazil. Arq Bras Oftalmol..

[b0350] Sindhuja K., Lomi N., Asif M.I., Tandon R. (2020). Clinical profile and prevalence of conjunctivitis in mild COVID-19 patients in a tertiary care COVID-19 hospital: A retrospective cross-sectional study. Indian J. Ophthalmol..

[b0355] Soltani S., Zandi M., Ahmadi S.E., Zarandi B., Hosseini Z., Akhavan Rezayat S. (2022). Pooled Prevalence Estimate of Ocular Manifestations in COVID-19 Patients: A Systematic Review and Meta-Analysis. Iran J. Med. Sci..

[b0360] Stephan S., Cohen F., Salviat F., Thevenin S., Devys J.M., Cochereau I. (2021). Evaluation of the impact of intensive care support for COVID-19 on the ocular surface in a prospective cohort of 40 eyes. Ocul. Surf..

[b0365] Wan K.H., Lui G.C.Y., Poon K.C.F., Ng S.S.S., Young A.L., Hui D.S.C. (2022). Ocular surface disturbance in patients after acute COVID-19. Clin. Exp. Ophthalmol..

[b0370] Wang Y., Yang S., Zhang Y., Zhang X., Jiang Y., Wang X. (2021). Symptoms of Dry Eye Disease in Hospitalized Patients with Coronavirus Disease 2019 (COVID-19). J. Ophthalmol..

[b0375] Wiersinga W.J., Rhodes A., Cheng A.C., Peacock S.J., Prescott H.C. (2020). Pathophysiology, transmission, diagnosis, and treatment of coronavirus disease 2019 (COVID-19): a review. J. Am. Med. Assoc..

[b0380] Wu P., Duan F., Luo C., Liu Q., Qu X., Liang L. (2020). Characteristics of ocular findings of patients with coronavirus disease 2019 (COVID-19) in Hubei Province, China. JAMA Ophthalmology..

[b0385] Zhong Y., Wang K., Zhu Y., Lyu D., Yu Y., Li S. (2021). Ocular manifestations in COVID-19 patients: A systematic review and meta-analysis. Travel Med. Infect. Dis..

[b0390] Zhou Y., Duan C., Zeng Y., Tong Y., Nie Y., Yang Y. (2020). Ocular Findings and Proportion with Conjunctival SARS-COV-2 in COVID-19 Patients. Ophthalmology.

[b0395] Zhou L., Xu Z., Castiglione G.M., Soiberman U.S., Eberhart C.G., Duh E.J. (2020). ACE2 and TMPRSS2 are expressed on the human ocular surface, suggesting susceptibility to SARS-CoV-2 infection. Ocul. Surf..

